# Mineral–Vitamin Complexes in Sheep Nutrition: Patent Analysis and Functional Evaluation for Pregnant Ewes and Lambs

**DOI:** 10.3390/molecules31060938

**Published:** 2026-03-11

**Authors:** Saltanat Baibatyrova, Akniyet Onerbayeva, Amirbek Sagyzbaev, Temirkhan Kenzhebaev, Zhazira Mukatayeva, Indira Kurmanbayeva

**Affiliations:** 1 Department of Chemistry, Abai Kazakh National Pedagogical University, Dostyk Ave., 13, Almaty 050010, Kazakhstan; saltabaibatyrova@gmail.com (S.B.); sagizbaev1996@gmail.com (A.S.); jazira-1974@mail.ru (Z.M.); 2 Head of Meat Sheep Breeding Department, Kazakh Research Institute of Livestock and Fodder Production, Zhandosov Str., Bldg. 51, Almaty 050035, Kazakhstan; kterdesh@mail.ru

**Keywords:** minerals, vitamins, ewes, lambs, feed additives

## Abstract

Natural pasture, the primary feed source in sheep production, often provides insufficient levels of essential minerals and vitamins required for proper metabolic regulation during pregnancy and early development. This study aimed to analyze patent developments of mineral and vitamin complexes (MVCs) for pregnant ewes and lambs and to evaluate the biochemical and molecular relevance of their components based on scientific evidence. A search of the World Intellectual Property Organization (WIPO) database using the keywords “vitamins for sheep” and “minerals for sheep” identified 120 patents related to sheep feed additives, including 23 specifically formulated for pregnant ewes and lambs. Comparative analysis revealed that calcium, selenium, iron, copper, cobalt, sodium, manganese, zinc, and vitamins A, D, and E were the most frequently included components. These micronutrients play critical roles in enzymatic activity, regulation of gene expression, antioxidant defense systems, and mineral homeostasis. In particular, zinc and selenium function as structural and catalytic cofactors for antioxidant enzymes such as superoxide dismutase and glutathione peroxidase, while vitamins A and D regulate cellular differentiation and calcium–phosphorus metabolism through transcriptional control mechanisms. Additionally, functional additives, including amino acids and plant-derived bioactive compounds, contribute to improved mineral bioavailability and modulation of metabolic pathways. The analyzed formulations demonstrate a consistent focus on correcting mineral deficiencies, enhancing antioxidant protection, and supporting metabolic adaptation during pregnancy and early postnatal development. Overall, the findings indicate that modern MVCs are rationally formulated to improve mineral utilization, physiological stability, and reproductive outcomes, highlighting their critical role in optimizing maternal health and offspring viability in sheep production systems.

## 1. Introduction

Sheep farming represents a strategically important sector of the agro-industrial complex. A substantial proportion of ewes are maintained under pasture and semi-pasture systems, where the mineral composition of available feed often does not meet the physiological requirements of the animals. Both pasture-based and stall-fed diets frequently fail to provide adequate levels of essential minerals and vitamins. Under grazing conditions, ewes—particularly lambs dependent exclusively on maternal milk—do not receive additional supplementation necessary to support critical physiological functions, which may negatively affect their survival, growth, and productive performance. Impaired physiological development and elevated mortality rates during the early postnatal period contribute to significant economic losses in sheep production. Therefore, the enrichment of animal feed with supplements containing biologically active compounds, including essential macro- and microelements, is necessary to meet the physiological requirements of lambs. Adequate nutritional support, particularly for pregnant ewes, plays a key role in ensuring proper embryonic and postnatal development, which is essential for the formation of healthy and productive breeding stock. Therefore, the objective of this review is to analyze patented mineral–vitamin complexes intended for pregnant ewes and lambs and to evaluate the biochemical and molecular relevance of their components based on scientific evidence.

A total of 226 patents were collected from the World Intellectual Property Organization (WIPO) database using the keywords “vitamins for sheep” and “minerals for sheep.” After applying a publication year filter (post-2006), thematic relevance screening, and selection criteria including the presence of feed or feed additives containing vitamins or minerals intended to improve sheep growth, productivity, and product quality, 23 patents specifically addressing formulations for pregnant ewes and lambs were selected for final analysis.

**Figure 1:**
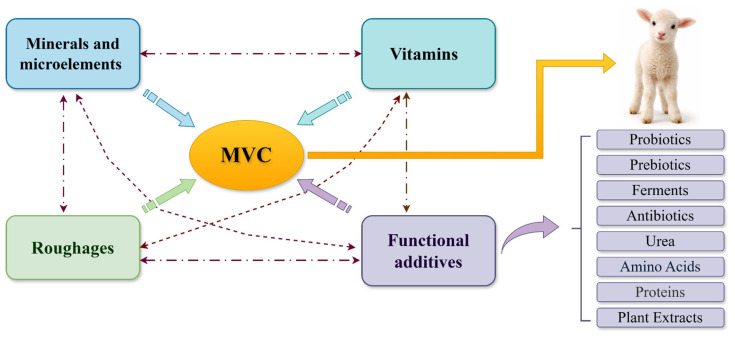
Structure of the mineral–vitamin complex formulated for pregnant ewes and lambs.

Analysis of 23 selected patents [1,2,3,4,5,6,7,8,9,10,11,12,13,14,15,16,17,18,19,20,21,22,23] demonstrated that their formulations comprise several key ingredient groups, including minerals, vitamins, functional additives (e.g., pharmaceutical agents, plant extracts, and microbial strains), and forage-based components. A schematic representation of the compositional structure of the mineral and vitamin complex (MVC) designed for pregnant ewes and lambs is presented in Figure 1.

Patent analysis was performed through an evaluation of the reported compositions, with all identified components classified into groups according to their functional roles, including minerals, vitamins, functional additives, and plant-derived ingredients. The frequency of inclusion of individual components in mineral–vitamin complexes was assessed to determine their prevalence and relevance in formulations intended for pregnant ewes and lambs.

Since the recommended inclusion levels of feed components are regulated by national standards and may vary among countries, quantitative dosage values were not considered as a primary criterion in the comparative analysis. Furthermore, patents do not always provide complete or precise dates, which represents an inherent limitation of patent-based studies.

The scientific rationale for selecting the components included in the analyzed complexes was evaluated based on relevant peer-reviewed publications addressing their biological functions, nutritional importance, and physiological effects in sheep, particularly in pregnant ewes and lambs.

## 2. Minerals and Microelements

### 2.1. Calcium

Calcium is the principal mineral component of feed additives, being included in 20 of the 23 analyzed patents [1,2,3,4,5,6,7,9,10,11,12,13,14,18,19,20,21,22,23]. The diversity of calcium sources is considerable and comprises calcium phosphate (Ca_3_(PO_4_)_2_) [3,4,5,10,12,13,14,17,20,21], calcium sulfate (CaSO_4_) [12,15], bone meal (36–58% Ca_3_(PO_4_)_2_) [1,2,5,6,7,13,14,18,19,21], calcium carbonate (CaCO_3_) [3,5,10,22,23], calcium hydrogen phosphate (CaHPO_4_) [3,4,5,10,12,13,14,17,20,21], and natural composites, such as stone powder, oyster shell whiting, ostreae testa pulverata, mountain flour, and calcium powders [9,11,13,20,21,22].

Calcium is the fundamental structural component of bone and teeth, and is also involved in neuromuscular transmission, muscle contraction, and blood coagulation processes [24]. Calcium homeostasis is maintained through continuous exchange between extracellular fluids and skeletal reserves, as well as through intestinal absorption and excretion via feces, urine, and milk. In addition, calcium is essential for mineral balance, growth, immune function, and proper development of young animals [25,26,27,28,29,30]. In sheep production systems, calcium deficiency is particularly common during pregnancy and lactation and may lead to osteodystrophy, reduced milk yields, and the birth of weak lambs [25,26,27,28,29].

Calcium absorption in ruminants occurs in both the rumen and small intestine and depends on dietary intake and physiological regulation. The rumen contributes significantly to calcium absorption when dietary calcium levels are high, whereas the small intestine serves as the primary site of regulated calcium absorption under low-calcium conditions due to increased calcium solubility and vitamin D-dependent active transport mechanisms [31,32,33,34].

Braithwaite (1983) demonstrated that during late pregnancy, intestinal calcium absorption alone is often insufficient to meet increased physiological demands, resulting in mobilization of calcium from skeletal reserves [26]. Adequate dietary calcium and phosphorus intake is therefore essential to restore mineral balance and support fetal skeletal development. Similarly, Brugger and Liesegang (2025) [25] reported that increased dietary calcium intake during late gestation and early lactation maintained mineral homeostasis through increased fecal excretion. However, excessive calcium intake was associated with altered bone metabolism and reduced average daily gain in lambs, suggesting that calcium oversupply may negatively affect postnatal growth [25].

Further evidence was provided by Ni et al. (2024), who demonstrated that optimal dietary calcium concentrations for growing sheep ranged from 0.73% to 0.89% of dry matter, maximizing growth performance and calcium retention, whereas excessive intake reduced mineral utilization efficiency [27]. The importance of balanced mineral supplementation during the transition period was also confirmed by Ataollahi et al. (2020), who reported that maternal supplementation with calcium and magnesium improved mineral status, antioxidant capacity, and growth performance of lambs [28].

Likewise, Abdelrahman (2008) showed that increased calcium intake during late gestation enhanced calcium concentrations in blood and colostrum and improved mineral status in newborn lambs [29].

In addition to its metabolic functions, calcium may influence gastrointestinal microbial activity. Khan et al. (2025) demonstrated that dietary calcium levels affect the composition and metabolic activity of the hindgut microbiota, including bacteria involved in short-chain fatty acid production and energy metabolism [30]. These findings indicate that calcium supplementation not only supports mineral homeostasis but may also indirectly enhance nutrient utilization and animal productivity.

The presence of calcium in various inorganic and organic forms in patented MVC formulations reflect its fundamental physiological importance and the need for precisely balanced supplementation to support skeletal integrity, metabolic stability, and productive performance in sheep.

### 2.2. Zinc

Zinc and its different forms are illustrated as additives on patented MVC formulae [1,2,3,4,6,7,8,10,11,12,13,14,16,17,20,21], which represent that the element is highly requested and recommended. Zinc ions are represented in the forms of zinc sulfate (ZnSO_4_) [3,4,8,10,11,16,20,21], zinc oxide (ZnO) [13], and zinc methionine [12]. Zinc plays an important role not only in well-known metabolic and immune processes, but also in ensuring reproductive potential, improving oocyte quality, and promoting embryo development [35].

According to a study by Xiwei Jin and co-authors, insufficient intake of Ca and Zn in sheep disrupts mineral and protein metabolism, causes liver damage and weakens physiological functions. However, even supplements do not always lead to the restoration of normal values due to low bioavailability and antagonism between the elements. For example, zinc can reduce copper absorption [36], which highlights the importance of balanced mineral support and early detection of hidden deficiencies.

In the work of G. Bellof et al., the effect of fattening intensity (up to a live weight of 18, 30, 45 and 55 kg) on the content of trace elements in various tissues was studied on 108 lambs of the German Merino breed [37]. An increase in nutrition levels led to an increase in daily gain and a decrease in energy expenditure per 1 kg of gain. At the same time, there was a decrease in Zn and Cu concentrations in tissues during high-intensity fattening, especially in females, while the Mn level often remained below the detection limit. These data confirm that the bioavailability and distribution of trace elements may vary depending on gender, growth stage, and feeding level.

Moreover, a study by K. Alijani and colleagues has demonstrated that the introduction of various forms of zinc (inorganic (ZnO), organic (zinc methionine) and nanoforms (nano-ZnO)) with particle sizes less than 100 nm into the diet of sheep has a positive effect on the digestibility of nutrients and body weight gain [38]. A particularly pronounced effect was observed when using organic and nanoscale forms: they provided a higher concentration of Zn in plasma and rumen contents, enhanced antioxidant activity (FRAP), reduced the level of ammonia and urea in the blood, and stimulated microbial protein synthesis [38]. Confirmation of these data is also contained in the study by Rodríguez-Maya et al., where lambs receiving combined Zn-Met + ZnO supplements showed the greatest weight gain, improved feed conversion and meat quality (marbling, softness of Longissimus dorsi muscles). This is associated with different mechanisms of absorption and bioavailability of zinc forms, as well as with its effect on regulatory proteins: ZnT7, ZnT8 and ZIP7 involved in insulin signaling pathways, glycemic control and differentiation of muscle cells. The organic form of zinc increased the content of arachidonic acid, while ZnO reduced the amount of visceral fat and saturated myristic acid [39]. The use of an organic form (zinc methionine) increases bioavailability and at the same time reduces the negative effects of high doses of inorganic salts, improving metabolism. Similar conclusions have been confirmed in experiments on calves conducted by Liu et al. [40]. It was found that the organic form of zinc (zinc proteinate, ZnPro) provides a more stable weight gain compared to ZnO, increases the immune response (IgG, IgM levels), reduces the frequency of diarrhea, and enhances the body’s antioxidant defense [40].

The mechanisms of zinc absorption in the gastrointestinal tract of ruminants, including sheep, largely determine its bioavailability [41]. According to D.L. Hampton, using radiolabeled zinc, it was found that upon oral administration, the element is absorbed along the entire length of the small intestine without pronounced localization in a certain section [41], which indicates that the absorption efficiency depends on the duration of contact of the chyme with the mucosa. In case of deficiency, it has been found that the posterior intestine has an extremely limited ability to absorb zinc and does not play a compensatory role. Thus, the main contribution to zinc absorption in ruminants is made by the small intestine, and the level of absorption is determined by the physiological state of the body and the availability of the trace element. The use of organic and nanoforms of Zn can significantly increase its digestibility and physiological effectiveness, especially with rational dosage selection and consideration of animal specificity [42].

According to the assessment of the European Food Safety Authority (2023) [42], the zinc chloride hydroxide monohydrate (IntelliBond Z) feed additive is recognized as safe for all animal species at the current level of use and does not pose a threat to consumers. On average, the Zn content in the product is about 57%, the main crystalline phase is simoncolleite. Simoncolleite or in another word tetrabasic zinc chloride with expected zinc oxide nanoscale particles (1–100 nm) has shown a paramount potential for animal nutrition not limited by only antibacterial property, but also higher bioavailability, growth promotion and enhancing the immune system. However, the dosage of zinc oxide nanoparticles is not decided to overcome toxicity since the mentioned compound with dosage of 40 mg per kilogram may have a danger for inner organ systems by irritating the genes and animal cells [43]. For users, the risk is mainly related to eye irritation and the possibility of dust inhalation, while skin sensitisation has not been confirmed. From an ecological point of view, the additive is acceptable in land-based farms and aquaculture, although data remain limited for marine systems. Research in the field of materials science expands the understanding of the biological role of simoncolleite. So, Li et al. (2019) [44] showed that simoncolleite, anchored to a poly (amino acid) matrix, is able to stimulate osteoblast proliferation and differentiation, increasing mineralization while inhibiting osteoclast formation. In addition, the material showed pronounced antimicrobial activity against Staphylococcus aureus and Escherichia coli. Thus, the prospects of simoncolleite-based compounds are determined not only by their role as a source of zinc, but also by their additional osteotropic and antimicrobial properties.

Thus, zinc is not just a structural trace element, but a multifunctional regulator that affects metabolism, growth, immunity, and product quality.

### 2.3. Selenium

Patent analysis indicates that selenium is included in MVC formulations in the forms of sodium selenite [3,8,10,11,13,19,20,21], nano-selenium [12], and selenium in unspecified chemical forms [1,2,6,7,14,16,17], reflecting the use of both conventional inorganic and emerging nano-scale selenium sources in practical feed supplementation strategies.

Selenium is an essential trace element involved in antioxidant defense, thyroid hormone metabolism, and immune regulation in ruminants. Its biological activity is primarily mediated through its incorporation into selenoproteins, including glutathione peroxidase and iodothyronine deiodinases [32]. These enzymes protect cells against oxidative damage by reducing hydrogen peroxide and lipid hydroperoxides and regulate thyroid hormone metabolism through the conversion of thyroxine (T_4_) to the biologically active triiodothyronine (T_3_). Adequate selenium supply therefore supports muscle integrity, immune competence, and reproductive performance in ewes. During pregnancy, selenium plays a particularly important role in protecting placental and fetal tissues from oxidative stress associated with rapid fetal growth and increased metabolic activity. Selenium deficiency reduces glutathione peroxidase activity and may result in oxidative damage, impaired fetal development, and increased neonatal mortality [24,45].

The safety and metabolic effects of inorganic selenium supplementation have been evaluated in several studies. Cristaldi et al. (2005) [46] demonstrated that dietary sodium selenite (Na_2_SeO_3_) supplementation at concentrations ranging from 0.2 to 10 ppm increased selenium concentrations in blood and tissues without inducing clinical signs of toxicity or histopathological alterations. These findings suggest that ruminants possess physiological mechanisms, including ruminal reduction of selenium to less bioavailable forms, that contribute to a relatively wide safety margin for inorganic selenium supplementation [46].

In contrast, nano-selenium exhibits higher biological activity and bioavailability. Shi et al. (2011) [47] reported that nano-selenium supplementation at 3.0 mg Se/kg dry matter optimized rumen fermentation, increased propionate production, enhanced fiber digestibility, and improved microbial protein synthesis. However, higher supplementation levels did not produce additional benefits, indicating that selenium efficacy depends strongly on dose and chemical form.

Selenium also functions synergistically with vitamin E as part of an integrated antioxidant defence system. Vitamin E protects cell membranes from lipid peroxidation, while selenium-dependent glutathione peroxidase removes reactive oxygen species and maintains intracellular redox balance [45]. Combined supplementation has been shown to improve antioxidant status, reduce stress-related physiological disturbances, and enhance metabolic stability in ewes (Chauhan et al., 2014 [48]; Chauhan et al., 2015 [49]), while Shakirullah et al. (2017) [50] reported reduced cortisol concentrations and increased antioxidant enzyme activity. Nutritional guidelines further emphasize that deficiency of either selenium or vitamin E increases susceptibility to oxidative stress and impairs immune, and reproductive function [24].

Overall, the evidence indicates that selenium supplementation is essential for maintaining antioxidant protection, metabolic stability, and reproductive performance in sheep. The presence of both sodium selenite and nano-selenium in patented MVC formulations reflects evolving nutritional strategies aimed at optimizing selenium bioavailability and physiological effectiveness. However, appropriate dose selection and consideration of interactions with vitamin E and other dietary factors remain critical to ensure both efficacy and safety, particularly during late gestation and early lactation.

### 2.4. Iron

In the analyzed patented MVCs, iron was present in three forms: ferrous sulfate [3,4,8,10,11,13,16,19,20,21], ferric methionine [12], and iron in unspecified chemical forms [1,2,6,7,14,17], as the patent authors did not provide detailed information on the compound type.

Iron is an essential trace element that plays a fundamental role in numerous physiological and biochemical processes in animals [32]. It is a key component of the heme prosthetic group in hemoglobin and myoglobin, enabling oxygen transport and storage, and serves as a cofactor for enzymes involved in mitochondrial electron transport, as well as catalase, myeloperoxidase, and cytochrome P450 systems. Iron deficiency leads to hypochromic microcytic anemia due to impaired hemoglobin synthesis and is often accompanied by reduced immune function.

The form and dose of iron significantly influence its biological effectiveness. Organic chelated forms of iron are particularly effective in preventing anemia and supporting hematopoiesis in lambs [51]. Asadi et al. (2022) [51] demonstrated that supplementation with iron-amino acid chelate (25 or 50 mg/day) in newborn lambs improved body weight gain and erythrocyte parameters, indicating correction of early-life iron deficiency. Iron supplementation also enhanced antioxidant enzyme activity (GPx, SOD, CAT) and improved metabolic status. An increase in insulin and thyroid hormones was noted with a decrease in glucose, which the authors attribute it to an improvement in metabolism. Based on the data obtained, 25 mg/day is the optimal dose, providing physiological benefits without disrupting mineral homeostasis.

Organic iron forms generally exhibit higher bioavailability and support antioxidant status when iron supply is adequate. However, supplementation in animals without iron deficiency may not produce additional physiological benefits, while excessive iron intake, even in chelated form, may promote oxidative stress and disrupt mineral balance, negatively affecting metabolic and organ function [52].

Parenteral iron supplementation has also demonstrated efficacy in correcting anemia [53]. Crilly and Plate reported that intramuscular administration of iron dextran (300 mg, 1.5 mL Uniferon™) to newborn lambs significantly increased hemoglobin concentrations and improved average daily gain, particularly in twin- and triplet-born animals. Similarly, goat kids receiving iron dextran showed significantly improved hemoglobin levels, although growth performance was not significantly affected. These findings indicate that iron deficiency anemia is common in young ruminants reared indoors and that parenteral iron supplementation effectively supports hematological status and growth. In contrast, dietary supplementation with ferrous sulfate at levels of 75 and 150 mg Fe/kg dry matter did not significantly affect dry matter intake, growth performance, or carcass characteristics in finishing lambs [54]. These results suggest that such supplementation levels maintain adequate iron status without negatively affecting rumen function, mineral balance, or metabolic stability, confirming their safety and physiological tolerability.

The iron forms identified in patented MVCs, including ferrous sulfate and ferric amino acid complexes, correspond to established nutritional approaches for improving iron bioavailability and preventing anemia in sheep [51,52].

### 2.5. Copper

Copper was identified in patented MVC formulations in the form of copper sulfate [3,4,8,10,11,13,16,20,21], nano-copper [12], and copper in unspecified forms [1,2,6,7,14,17]. The inclusion of both inorganic and nano-scale copper sources reflects current technological approaches aimed at optimizing copper bioavailability and physiological efficacy in ewes.

Copper is an essential trace element involved in iron metabolism, mitochondrial respiration, antioxidant defense, connective tissue formation, and pigmentation. Both copper deficiency and excess disrupt physiological homeostasis in ruminants [55,56]. Field observations in South Australia have demonstrated that copper deficiency results in reduced fertility, increased lamb mortality, and decreased wool quality leading to significant economic losses. Correction strategies, including injections, intraruminal boluses, mineral supplements, and suspensions, have been shown to increase hepatic copper reserves; however, their effectiveness is strongly influenced by dietary antagonists such as molybdenum, sulfur and iron, which reduce copper bioavailability and limit the efficacy of supplementation programs [56].

In contrast, excessive copper intake may result in toxic effects, particularly when administered in highly bioavailable forms. Song et al. [57] demonstrated that dietary nano-copper (≥99.9%, 150 to 200 nm) supplementation in ewes induced hematological suppression, increased hepatic enzyme activities (aspartate aminotransferase, alanine aminotransferase), elevated lipid peroxidation, and reduced antioxidant enzyme activity, indicating oxidative stress and hepatic dysfunction. These findings highlight the narrow safety margin of copper supplementation and the need for careful dose regulation, especially when using nano-scale formulations.

Conversely, appropriate copper supplementation improves physiological and immune functions. Min et al. reported [58] that supplementation with nano-Cu_2_O or CuSO_4_ in copper-deficient sheep increased blood copper levels, improved hematological parameters, enhanced antioxidant enzyme activity, and reduced oxidative stress markers, with nano-Cu_2_O demonstrating greater bioavailability and efficacy. Similarly, Senthilkumar et al. [59] demonstrated that dietary copper supplementation (7–14 mg/kg dry matter) in the form of copper sulfate or organic copper proteinate (copper proteinate is copper bound to protein), particularly in organic proteinate form, enhanced humoral and cellular immune responses, increased antioxidant enzyme activity, and improved overall immune competence. Organic copper forms showed superior immunomodulatory effects, while inorganic copper sulfate demonstrated stronger effects on specific antioxidant enzyme activity.

These findings confirm that copper plays a critical role in maintaining metabolic stability, antioxidant defense, and immune function in ewes. However, both deficiency and excess can adversely affect animal health and productivity. Therefore, effective copper supplementation strategies must consider the chemical form, dosage, bioavailability, presence of antagonistic elements, and the physiological and nutritional status of the animals. The inclusion of copper in patented MVC formulations reflects its essential role in supporting metabolic and immune functions, although the use of highly bioavailable forms, particularly nano-copper, requires careful evaluation to avoid toxicity.

### 2.6. Manganese

Manganese is reported in patented MVCs in the form of manganese sulfate (MnSO_4_·H_2_O) [3,8,10,11,13,16,20], manganese methionine (Mn-Met) [6,7,12], and manganese in an unspecified chemical form [1,2,14]. Manganese (Mn) plays an important role in reproductive function, skeletal development, and mineral metabolism in sheep. The inclusion of organic Mn sources in mineral–vitamin complexes is often justified by their potentially higher bioavailability compared with inorganic salts. Hidiroglou et al. (1978) evaluated the effects of dietary manganese levels on reproductive performance of ewes [60]. Manganese was supplied in inorganic form as manganese sulfate monohydrate ${(MnSO}_{4}$·$H_{2}O)$ at 60 ppm, whereas the control diet contained 8 ppm Mn. Diets were fed for five months prior to breeding and throughout gestation. Ewes receiving the low-manganese diet required a higher number of services per conception, indicating impaired reproductive efficiency under marginal Mn supply. Alterations in tissue mineral composition were also observed. In particular, changes in calcium distribution suggested that manganese deficiency may interfere with mineral interactions necessary for normal fetal development [60].

These results indicate that manganese exerts not only a structural but also a regulatory role through its involvement in metalloenzymes and interactions with other trace elements that are critical for reproduction.

Study [61] compared inorganic manganese sulfate (MnSO_4_) and organic manganese glycinate chelate (MnGly) supplementation in lamb diets at 150 mg/kg. While no significant differences were observed in nutrient digestibility or Mn distribution, manganese glycinate improved fiber utilization and increased Mn availability to rumen microorganisms [62]. Additional evidence from other livestock species supports the physiological relevance of organic Mn sources. In sows, organic manganese improved birth weight, pre-weaning growth, and milk composition [60]. Similarly, in laying hens, organic Mn sources enhanced shell quality and productivity compared with manganese oxide [63]. Although these studies were not conducted in sheep, they support the concept that manganese source can influence reproductive and developmental outcomes across species.

Overall, available evidence indicates that manganese plays a critical role in reproductive performance and trace element metabolism in ewes and lambs, while the choice of Mn source may influence biological efficiency under certain dietary conditions.

### 2.7. Cobalt

Cobalt is represented in patented MVCs in the form of cobalt [1,2,6,7,14,16,17] and cobalt chloride (CoCl_2_·6H_2_O) [3,8,10,11,12,13,20,21]. Cobalt is an essential trace element in sheep nutrition due to its role in ruminal vitamin B_12_ synthesis, which is required for energy metabolism and growth. Cobalt deficiency impairs feed intake, growth performance, and metabolic efficiency in lambs [64,65]. In a study by Rothery et al., using radiocobalt, it was demonstrated that cobalt is actively distributed in the tissues and gastrointestinal contents of ruminants, confirming its essential role in ruminal cobalamin (vitamin B_12_) synthesis [66]. Subsequent studies have shown that cobalt deficiency is associated with reduced activity of vitamin B_12_-dependent enzymes. Kennedy et al. reported that lambs with Co–B_12_ deficiency exhibited decreased activity of methionine synthase and methylmalonyl-CoA mutase, leading to the accumulation of methylmalonic acid and the formation of abnormal fatty acids [67]. In a study by Vellema et al., intraruminal cobalt pellets (long-acting boluses) were used to provide a sustained supply of cobalt to the rumen. This supplementation improved cellular immune responses following vaccination and reduced gastrointestinal nematode burden, highlighting the important role of cobalt–vitamin B_12_ status in the development of immune competence in sheep [68].

Aliarabi et al. investigated the effects of a sustained-release bolus containing zinc, selenium, and cobalt administered six weeks before lambing on the productivity and metabolic status of Mehraban sheep and their lambs [69]. Bolus supplementation improved mineral status, as evidenced by increased plasma concentrations of Zn, Se, and vitamin B_12_ in both ewes and lambs, as well as higher milk mineral content. Lambs born to supplemented ewes showed greater birth and weaning weights, higher average daily gain, and reduced mortality and incidence of white muscle disease. In addition, supplementation enhanced antioxidant status, indicated by increased glutathione peroxidase activity, and improved metabolic and endocrine parameters. These findings demonstrate that intraruminal administration of trace elements in sustained-release form during late pregnancy improves maternal and neonatal mineral status and enhances lamb growth and survival [69].

Natural cobalt supplementation sources can also be effective. For instance, feeding willow leaves, which contain high cobalt concentrations, significantly increased plasma vitamin B_12_ levels in lambs compared with unsupplemented animals, confirming the importance of dietary cobalt for maintaining adequate vitamin B_12_ status [64].

Cobalt supplementation is particularly important in grazing systems, as pasture cobalt concentrations often do not meet dietary requirements. Supplementation improves vitamin B_12_ status and enhances growth performance, especially in young and weaned lambs.

### 2.8. Magnesium

Magnesium was reported in MVC patents in the form of magnesia [16], MgO [13,19], and magnesium sulfate [8,10,19,20]. Magnesium plays an important role in neuromuscular function, rumen activity, and mineral metabolism in sheep, particularly during periods of increased physiological demand.

Chester-Jones et al. [70] evaluated the effects of elevated dietary magnesium levels in lambs fed diets containing MgO at concentrations of 0.2, 0.6, 1.2, and 2.4%. Increasing magnesium intake altered Mg, Ca, and P metabolism and promoted magnesium accumulation in tissues. Prolonged intake above 1.2% negatively affected productivity, whereas approximately 0.5% Mg was considered a safe upper level under the studied conditions. In addition to dosage effects, sheep exhibit the ability to regulate mineral intake according to physiological needs. Studies have shown that sheep can distinguish between magnesium sources and adjust their intake depending on mineral status, suggesting a mechanism of nutritional self-regulation [71].

These findings indicate that both magnesium dose and bioavailability are important determinants of mineral metabolism and physiological stability in sheep. This is particularly relevant for pregnant and lactating ewes, in which magnesium imbalance may disrupt metabolic homeostasis and interfere with calcium and potassium metabolism.

### 2.9. Potassium, Sodium

Potassium and sodium are the main functional intracellular cations in mammals. The inclusion of these components is aimed at maintaining the acid-base balance and optimal functioning of the scar. Sodium is represented in patented MVCs in the form of salts of sodium bicarbonate [3,4,5,13,17,22], sodium sulfate [3,13,20,22] and sodium chloride [1,2,3,6,7,12,15,18,19,22,23]. Potassium is found in patents in the form of potassium sulfate [19] and potassium iodide [3,8,10,11,12,13,20,21]. Ruminants commonly consume potassium in excess of their physiological requirements, particularly under grazing conditions, and are well adapted to its metabolism and regulation. Potassium and sodium are major cations in rumen fluid and saliva and play essential roles in maintaining osmotic balance and rumen function. Cations in the scar fluid significantly affect digestion; when sodium is deficient, potassium can partially replace it in saliva [72].

Phillips et al. identified a critical dietary potassium concentration of approximately 30 g/kg dry matter for non-lactating Welsh Mountain ewes [73]. Potassium intake above this level was associated with reduced magnesium absorption and retention, increasing the risk of hypomagnesemia, and may also impair calcium absorption [74]. Conversely, inadequate potassium levels may disrupt intracellular calcium balance and increase the risk of hypocalcemia. Lipecka et al. evaluated the effects of genetic variation in blood potassium levels in ewes and lambs and found no significant differences in growth performance or wool yield between high-potassium (HK) and low-potassium (LK) phenotypes [75]. However, ewes with the HK phenotype exhibited significantly reduced fertility and reproductive performance. These findings suggest that maintaining optimal potassium levels is essential for mineral balance and reproductive function in sheep.

Dietary NaHCO_3_ supplementation has been associated with improvements in carcass characteristics and meat quality in lambs, likely due to its role in maintaining acid–base balance and optimizing rumen function [76]. Also incorporation of NaHCO_3_ to a diet fed to weaner lambs improved feed intake and fiber digestibility [77].

### 2.10. Other

Natural mineral matters (in [9,18]), including zeolite (in [10,12]) and bentonite (in [3]), are used in patented formulations due to their high adsorption capacity, enabling binding of mycotoxins and other toxic compounds [78]. Their inclusion in animal diets has been associated with improved detoxification, liver function, immune response, and productivity.

Organic rare earth elements (REEs) are incorporated in patented MVCs [9,18]. Supplementation of animal feeds with rare earth elements has been practiced in China for several decades. Following restrictions on antibiotic growth promoters, REEs have attracted increasing attention as potential alternative feed additives. Numerous studies have reported that appropriate dietary levels of REEs can improve animal health, enhance body weight gain, increase feed conversion efficiency, and improve milk and egg production [79]. Chelated forms such as REE–citrate, have been investigated as functional feed additives in sheep due to their ability to enhance rumen microbial activity, improve nutrient digestibility, and promote growth performance [80].

Iodine is represented in patent developments in the form of potassium iodide [14,16,17]. Its use is due to the need to prevent iodine deficiency in pregnant queens and ensure the normal development and functional activity of the thyroid gland in lambs [81,82]. Chromium is an essential trace element involved in glucose and lipid metabolism, with trivalent chromium (Cr^3+^) enhancing insulin signaling. Supplementation with nano-chromium picolinate has been shown to improve physiological adaptation to heat stress in sheep by reducing rectal temperature and respiration rate [83].

**Figure 2:**
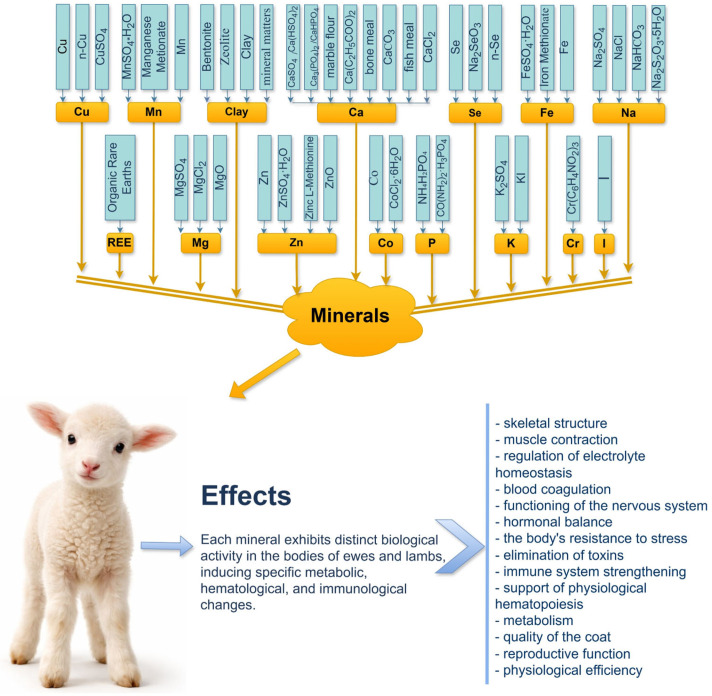
Mineral components in patented MVCs for ewes and their physiological functions.

The diagram (Figure 2) shows that patented MVC formulations incorporate a wide range of essential macro- and microelements in various inorganic and organic forms. These minerals play critical roles in maintaining metabolic homeostasis, supporting physiological development, enhancing immune function, and improving productivity in sheep, particularly during periods of increased physiological demand such as pregnancy, lactation, and early growth.

These mineral components contribute to essential biological processes, including skeletal development, muscle contraction, electrolyte balance, hematopoiesis, immune function, hormonal regulation, and metabolic homeostasis. Adequate mineral supplementation supports stress resistance, detoxification, reproductive performance, wool quality, and overall physiological efficiency in ewes.

## 3. Vitamins

### 3.1. Vitamins E, A, D

The analysis of patent sources demonstrated the widespread use of multicomponent (multivitamin) formulations. Multivitamins [3,4,6,7,14,15,19,20,21] are most frequently reported. In several patents, these complexes include combinations of fat- and water-soluble vitamins. For example, patent [4] contains vitamins A, K_3_ and E, whereas compound vitamin formulations described in patents [6,7] include vitamin E together with B_2_, B_6_, B_12_ and vitamin C. In some patents, the presence of vitamins is mentioned without specifying their exact composition, for instance [3,20], where the vitamin complex is indicated but the individual vitamins are not specified. In another formulation [14], the vitamin mixture consists of vitamin A (50%), vitamin B complex (20%), vitamin D (4%), vitamin E (6%) and vitamin K (20%). Individual vitamins are also included in the formulations, including vitamin A [5,8,10,11,12,13,14,15,16,17], vitamin D_3_ [5,8,10,11,12,13,14,15,16,17], vitamin E [1,2,7,8,10,11,12,13,14,16,17], vitamin K [11,14], and vitamin C [1,2,7]. B-group vitamins are also commonly included: vitamin B_1_ [10,14,17], vitamin B_2_ [1,2,7], vitamin B_6_ [1,2,7], vitamin B_12_ [1,2,7,21], as well as biotin [10], niacin [8], nicotinamide [10], and nicotinic acid [17]. The diversity of these components reflects the multifunctional role of vitamins in regulating metabolism, immune responses, growth and reproductive performance in sheep.

The inclusion of vitamins A, D, and E in MVCs reflects their essential physiological roles in sheep. Vitamin A regulates epithelial integrity, immune function, and steroid hormone synthesis, which are critical for reproductive performance and embryonic development [84]. Vitamin E functions as a lipid-soluble antioxidant that protects cellular membranes from oxidative damage and supports neonatal survival and reproductive efficiency in ewes [85]. Vitamin D regulates calcium and phosphorus metabolism and is therefore essential for skeletal mineralization, fetal development, and maintenance of mineral homeostasis [86]. These physiological functions explain why vitamins A, D, and E are commonly incorporated into MVC formulations for pregnant ewes and lambs. The effects of vitamin supplementation on reproductive and physiological performance in sheep are context-dependent. Hashem et al. (2016) demonstrated that supplementation with vitamins A or C under heat stress conditions improved hematological parameters, increased fertility, and enhanced steroid hormone concentrations in Rahmani sheep [84].

In contrast, Raasch et al. (1998) found that parenteral administration of vitamins A and E did not significantly improve fertility or embryo survival under standard management conditions [87]. Similarly, Donnem et al. (2015) reported that vitamin E supplementation reduced stillbirth rates primarily in ewes carrying multiple fetuses, while general supplementation produced inconsistent benefits [85]. Kott et al. (1998) also observed reduced early lamb mortality with vitamin E supplementation, although the magnitude of the effect varied depending on environmental and management factors [88]. Combined supplementation with selenium and vitamin E improved pregnancy rates and mating success in synchronized ewes [89], while neonatal vitamin A administration enhanced spermatogenesis and reproductive potential in male offspring [90]. Furthermore, Efe et al. (2023) demonstrated that supplementation with selenium and vitamins A, D, and E partially improved reproductive performance in sheep fed micronutrient-deficient diets, although responses varied among parameters [91].

These findings indicate that vitamins A and E do not universally enhance fertility but exert the greatest benefits under conditions of deficiency, oxidative stress, heat stress, multiple pregnancy, or mineral imbalance. Under nutritionally adequate conditions, additional supplementation may produce limited improvements. Therefore, the inclusion of vitamins A and E in MVC formulations should be based on physiological requirements, dietary adequacy, and environmental stress conditions.

Vitamin D status in sheep is influenced by both dietary intake and endogenous synthesis. Nemeth et al. (2017) demonstrated that lambs and goat kids fed vitamin D-deficient diets maintained circulating 25-hydroxyvitamin D concentrations through UV-B-induced skin synthesis, although species-specific differences in mineral metabolism and bone parameters were observed [86]. Zhou et al. (2019) reported that maternal vitamin D status varied among breeds and was positively associated with lamb birth weight, suggesting an important role in fetal development [92]. However, Kohler et al. (2013) found that increased ultraviolet exposure did not significantly improve vitamin D status in lactating sheep, indicating a greater dependence on dietary sources [93].

Overall, these findings suggest that vitamin D plays a critical role in skeletal development and fetal growth rather than directly influencing fertility. Its availability depends primarily on dietary supply, mineral balance, and physiological stage, while ultraviolet radiation provides a supplementary source. Accordingly, the inclusion of vitamin D in MVC formulations is essential to support calcium metabolism, skeletal integrity, and neonatal development, particularly under intensive production systems or limited sunlight exposure.

### 3.2. B Vitamins

Patented vitamins identified in MVCs include thiamine (B_1_), riboflavin (B_2_), niacin (B_3_), pantothenic acid (B_5_), Pyridoxine (B_6_), biotin (B_7_), folate (B_9_), and cobalamin (B_12_). These vitamins function primarily as cofactors for enzymes involved in carbohydrate, lipid, and protein metabolism, supporting energy production, rumen microbial activity, and nutrient utilization in sheep. Under normal physiological conditions, rumen microorganisms synthesize substantial amounts of B-group vitamins; however, supplementation may become necessary during periods of increased metabolic demand, rapid growth, pregnancy, or impaired rumen function. Among B-group vitamins, vitamin B_12_ has particular importance in ruminants due to its cobalt-dependent microbial synthesis and essential role in propionate metabolism. As a cofactor for methylmalonyl-CoA mutase, vitamin B_12_ enables the conversion of propionate to glucose via gluconeogenesis, which is critical for maintaining energy balance, especially during late pregnancy and early lactation.

Rickard and Elliot [94] demonstrated that metabolic function depends on the biologically active forms of cobalamin, as inactive analogues can impair propionate metabolism despite adequate total vitamin concentrations.

Similarly, Marca et al. (1996) reported that parenteral vitamin B_12_ supplementation did not improve growth or vitamin status in lambs with adequate cobalt intake, emphasizing the importance of endogenous microbial synthesis [95]. Gruner et al. (2009) [96] further demonstrated limited retention of exogenous vitamin B_12_ in young lambs, indicating that supplementation is most effective when cobalt deficiency or rumen dysfunction limits microbial synthesis [96]. Supplementation with B-vitamin complexes has been shown to improve metabolic and physiological responses during periods of increased demand. Asadi et al. (2024) [97] reported that administration of a B-vitamin complex increased plasma vitamin concentrations, improved feed intake, enhanced thyroid hormone activity, and strengthened immune responses in small ruminants during the periparturient period. Improved glucose status and reduced insulin concentrations indicated enhanced metabolic adaptation and energy balance.

Other B-group vitamins contribute to metabolic regulation and tissue integrity under specific physiological and nutritional conditions. Niacin has been shown to influence rumen fermentation and improve metabolic efficiency by modulating microbial activity and energy metabolism [98]. Choline chloride is a quaternary ammonium salt with choline cation and chloride anion (C_5_H_14_ClNO), identified in patented formulations [21], serves as an important methyl donor involved in lipid metabolism, liver function, and cellular membrane integrity. As a bioavailable source of choline (vitamin B_4_), it contributes to improved growth performance and metabolic stability, particularly during periods of increased physiological demand [99,100].

Biotin (vitamin B_7_), also identified in patented formulations [10], plays a key role in keratin synthesis, energy metabolism, and hoof health. Bampidis et al. (2007) [101] demonstrated that long-term biotin supplementation (0.21, 3.26 and 5.25 mg/ewe/day) significantly improved hoof integrity and locomotion in sheep with chronic lameness, indicating enhanced keratin formation and structural repair. These effects were dose-dependent and consistent with the slow regeneration rate of hoof tissue. Peterson et al. (2004) [102] further demonstrated that high-concentrate diets may reduce effective biotin availability despite rumen microbial synthesis.

Overall, these findings demonstrate that B-group vitamins play essential roles in enzymatic reactions, energy metabolism, and physiological adaptation in sheep. Although endogenous rumen synthesis provides a substantial supply under normal conditions, targeted supplementation in MVC formulations is beneficial during pregnancy, rapid growth, metabolic stress, or impaired rumen function. The inclusion of B-group vitamins in MVCs reflects their biochemical importance in maintaining metabolic homeostasis, supporting immune function, and optimizing animal productivity [102].

The inclusion of B-group vitamins in patented MVC formulations reflects their fundamental role in energy metabolism and rumen function, particularly under conditions where endogenous microbial synthesis may be insufficient to meet physiological demands.

### 3.3. Vitamin K

In the analyzed patented MVCs, vitamin K was identified relatively infrequently, being present in formulations [4,11,14,21]. Despite its limited inclusion, vitamin K plays an essential physiological role in bone metabolism and blood coagulation in sheep. It is required for the activation of vitamin K-dependent proteins, including osteocalcin, which is critical for proper bone mineralization and skeletal integrity. Insufficient vitamin K supply may impair bone mineralization, leading to reduced bone strength and an increased risk of skeletal abnormalities [103]. Experimental evidence supports the importance of vitamin K in skeletal metabolism. Roy and Lall (2007) demonstrated that vitamin K deficiency was associated with reduced bone mineral density and altered bone structure, indicating impaired calcium and phosphorus utilization and compromised skeletal health [103].

In addition to its role in bone metabolism, vitamin K is essential for blood coagulation. A congenital disorder affecting vitamin K-dependent coagulation factors has been reported in Rambouillet lambs, resulting in severe hemorrhage and increased neonatal mortality due to reduced activity of clotting factors II, VII, IX, and X [104]. The absence of dietary antagonists and only partial response to supplementation suggested a genetic defect affecting vitamin K metabolism or γ-glutamyl carboxylase activity. Pedigree analysis confirmed autosomal recessive inheritance, indicating that vitamin K-related disorders may arise from both nutritional and genetic causes.

Vitamin K may also influence early embryonic development. Sefid et al. (2017) demonstrated that vitamin K_2_ supplementation in in vitro embryo culture improved blastocyst formation, embryo quality, and mitochondrial function while reducing oxidative stress [105]. Although these findings suggest potential antioxidant and metabolic roles of vitamin K during early development, their relevance to practical feeding systems requires further investigation.

Overall, vitamin K contributes to skeletal development, blood coagulation, and early developmental processes in sheep. Its relatively infrequent inclusion in patented MVC formulations may be explained by partial endogenous synthesis by rumen microorganisms and the limited availability of applied nutritional studies in sheep. Nevertheless, under conditions of increased physiological demand or impaired rumen function, vitamin K supplementation may contribute to improved metabolic and reproductive outcomes.

The inclusion of vitamin K in patented MVC formulations, although limited, reflects its important but often overlooked role in supporting skeletal integrity, coagulation function, and developmental processes in sheep.

Overall, analysis of the available literature indicates that A-, D-, E-, B-group vitamins and vitamin K play essential but complex roles in regulating reproductive function, growth, and metabolic stability in sheep. Their effects are not universal and depend largely on nutritional status, breed characteristics, physiological stage, management conditions, and diet composition. Vitamins A and E primarily exert supportive and protective functions. Under conditions of deficiency or physiological stress—such as heat stress, early lactation, or multiple pregnancy—they contribute to maintaining reproductive performance, reducing neonatal mortality, and supporting hormonal and immune function. However, under nutritionally adequate conditions, additional supplementation often provides limited or inconsistent benefits.

Vitamin D is more strongly associated with skeletal development, intrauterine growth, and lamb birth weight than with fertility itself. Its status in sheep is determined primarily by dietary intake and mineral balance, while ultraviolet exposure provides only a supplementary source. B-group vitamins, particularly vitamin B_12_, are closely linked to cobalt availability and rumen microbial activity. Supplementation with vitamin B_12_ in the absence of deficiency generally produces minimal physiological effects, whereas continuous and balanced supply of B-group vitamins during metabolically demanding periods supports energy metabolism, immune function, and overall productivity. These findings emphasize the importance of adequate cobalt nutrition and functional assessment of vitamin B_12_ status.

Vitamin K is available evidence confirms its important role in bone mineralization, blood coagulation, and early embryonic development. Taken together, these findings indicate that vitamin supplementation strategies in sheep should be based on physiological requirements, dietary adequacy, and environmental conditions to ensure optimal metabolic function and reproductive performance.

## 4. Functional Additives

An analysis of patented mineral and vitamin complex formulations indicates that some are designed to enhance biological activity by including of probiotics, prebiotics, fermented products, urea, amino acids, and various phytocomponents, which may provide functional benefits beyond basic nutritional supplementation. However, several MVCs also contain antibiotics, such as monensin sodium [1,2,3,6,7,16,19] and bacitracin zinc [1,2,6,7,19], whose use is subject to strict regulatory control and is prohibited in the European Union [106,107]. The inclusion of such compounds reflects substantial regional disparities in regulatory frameworks and feeding practices and raises concerns regarding safety, antimicrobial resistance, and regulatory compliance.

Probiotics [23], including *Bacillus subtilis* and *Bacillus licheniformis* [21], as well as *Lactobacillus* and *Bifidobacterium species* [14], L-lactic acid (Pfanstiehl) [21], and prebiotics [21], were incorporated into MVC formulations to improve intestinal function and nutrient utilization. In addition, Rumensin™ (a registered premix containing monensin) was included in patents [3,18]. Pfanstiehl, identified as L-2-hydroxypropionic acid (L-lactic acid), is a biologically active organic acid that can be metabolized by the host organism and has been reported to support gut microbiota balance and promote intestinal health [108]. Notably, a component referred to as *Bacillus* titanium–zinc was identified in four patents [1,2,6,7], although this term has not been described in the peer-reviewed scientific literature. According to the patent descriptions, this component exerts growth-promoting effects in ewes.

Experimental evidence supports the use of probiotics and functional feed additives as potential alternatives to antibiotics in ruminant nutrition [106,107]. Supplementation with *Bacillus subtilis* (strain C-3102) has been shown to enhance fiber fermentation, increase microbial protein synthesis, optimize volatile fatty acid profiles, and improve body weight gain without increasing feed intake [109]. Similarly, probiotic formulations based on *Bacillus licheniformis* and *Saccharomyces cerevisiae* demonstrate effects comparable to monensin, improving feed efficiency, growth performance, and hormonal and antioxidant status [110]. Additional studies have reported that probiotics improve intestinal morphology, enhance amino acid and vitamin metabolism, modulate immune function, and promote beneficial shifts in microbiota composition, ultimately supporting improved metabolic efficiency and productivity [111,112]. However, the effectiveness of probiotic and functional additives depends on strain specificity, dosage, and diet composition, highlighting the importance of evidence-based optimization for practical applications [111].

Prebiotic components identified in patented MVC formulations include oligosaccharides [21], orange peel [21], and tangerine peel [14]. Oligosaccharides function as selective substrates for beneficial microorganisms, promoting growth performance, immune function, nutrient digestibility, and antioxidant status in sheep [113,114,115]. Citrus by-products, including orange and tangerine peels, represent valuable functional feed ingredients, as rumen microorganisms are capable of fermenting fibrous plant materials and utilizing their bioactive compounds, including flavonoids and polyphenols, which may contribute to improved gut health and metabolic stability [116].

Composite microbial fermentation mixtures were described in patents [1,2,6,7], consisting of *Bacillus subtilis*, *Bifidobacterium*, and *Streptococcus intermedius* in a 1:1:1 ratio. In addition, several patents reported the inclusion of exogenous enzymes, such as cellulase [10,12,14] and protease [10], which may enhance fiber degradation and protein utilization, thereby improving feed efficiency.

In the analyzed patents, amino acid additives were identified in formulations [14,23] as compound amino acid mixtures containing lysine (40%), methionine (46%), and threonine (14%) on a weight basis [14]. Methionine was also included as an individual component in patents [13,21], while lysine was present in patents [13,19,21]. In addition, derivatives related to glutamic acid and propylhomoserine were reported in patent [17]. Supplementation with rumen-protected methionine increased in milk yield and protein synthesis in dairy sheep [117], while combined lysine and methionine supplementation improved milk production, casein content, and lamb growth performance [118]. Zhang et al. (2025) [119] demonstrated that an optimal lysine-to-methionine ratio of 1:1 improved jejunal short-chain fatty acid concentrations by modulating the microbial community and regulating key metabolic pathway such as pantothenate, pantothenic acid, phosphoenolpyruvate, isodeoxycholic acid, and 3-dehydrocholic acids, thereby enhancing intestinal barrier function.

Yeast was included as a functional additive in several patents [12,14,17,19], due to its ability to enhance ruminal fiber degradation, stabilize microbial populations, and improve nutrient digestibility and feed efficiency [120,121].

Several patents also included less commonly reported functional components, such as urease inhibitors [16], aminophylline, which is known as bronchodilators [21], mercaptamine (cysteamine) [9], and casein [21]. Research indicates that although urease inhibitors can improve nitrogen utilization efficiency and reduce nitrogen losses, their effects on growth performance in sheep fed urea-containing diets remain inconsistent [122,123]. Aminophylline belongs to a group of medicines known as bronchodilators. Cysteamine is a biologically active aminothiol involved in coenzyme A metabolism and has been associated with improved growth performance through modulation of protein metabolism and endocrine regulation [124]. Casein plays a key role in protein nutrition and in the transport of calcium and phosphorus, which are essential for neonatal development [125].

Additional nutritional and functional ingredients identified in patented formulations include cod liver oil [4,5], starch [16], molasses [3,22], mulberry leaves [23], hawthorn [9,14,20], pine needle meal [8], and glycoconjugates [21]. Cod liver oil provides essential omega-3 fatty acids and fat-soluble vitamins, supporting immune function and reproductive performance. Starch serves as a readily available energy source and improves feed processing and palatability. Molasses enhances feed intake and provides fermentable carbohydrates to support rumen microbial activity [126]. Mulberry leaves and hawthorn contain bioactive polyphenols with antioxidant and immunomodulatory properties, while pine needle meal contains phytochemicals (tannins, essential oils, polyphenols and flavonoids) with potential antioxidant and antiparasitic effects [127]. Glycoconjugates are complex carbohydrates with proteins or lipids. It may contribute to gastrointestinal integrity, immune modulation, and metabolic regulation, supporting overall animal health and productivity [128,129]. Table 1 presents functional additives from mineral–vitamin complex formulations.

Functional additives are widely used in MVC formulation for pregnant ewes and lambs. Functional supplements affect the microbiota by improving the absorption of vitamins and minerals. They not only improve the digestibility of feed and the bioavailability of minerals, but also stimulate the immune system and increase the resistance of animals to stress and disease.

## 5. Feed Additives (Roughages)

In the analyzed patented MVCs, plant-derived feed ingredients constitute approximately one-quarter of the total formulation, reflecting their role as carriers, nutrient sources, and functional components. These formulations include a wide range of cereal grains and roughage-derived materials, such as corn [1,2,3,4,5,6,7,8,12,14,15,17,19,20,22,23], wheat [1,2,3,4,6,7,9,12,13,14,15,17,18,23], barley [15], rice [11,12,15,17,18], and forage sources including green hay [1,2,6,7,9,12,17,23], alfalfa [14,17,20,21], clover [15,23], and ryegrass powder [14]. These ingredients are incorporated in various processed forms, including flour, straw, silage, and meal. Some patents describe the inclusion of straw powder without specifying the plant origin [9,10,18]. Although the original particle size of roughages varies, mechanical processing during feed preparation reduces particle size, improving mixing uniformity and digestibility. Dried distillers grains with solubles (DDGS), commonly derived from corn, were also identified in several patented formulations [8,12,17,20,22]. DDGS are rich in protein, energy, and digestible fiber, and meta-analysis data [130] indicate that inclusion levels of up to 20% in sheep diets improve growth performance, nutrient digestibility, carcass characteristics, and meat quality.

Oilseed by-products represent another important group of ingredients in patented MVC formulations. These include flaxseed cake [1,2,6,7,17], sesame cake [1,2,6,7,9], sunflower cake [1,2,6,7,12,17,18,19], pumpkin seed by-products [15], detoxified castor cake [14], and saccharified liquid filter residue [20]. These materials provide protein, essential fatty acids, and bioactive compounds that support growth and metabolic function. Plant-derived agricultural residues were also widely incorporated, including Chinese cabbage leaves [1,2,6,7,23], peanut sprouts and vines [1,2,4,5,6,7,23], sweet potato vines [1,2,6,7,23], palm meal [12], rapeseed dregs [3,4,22], soybean and legume residues [4,5,7,8,15,17], cotton by-products [15], and bamboo-derived materials [4,5]. These materials serve as cost-effective sources of protein, fiber, and bioactive compounds. Studies have demonstrated that cotton by-products can be effectively used in sheep diets without negative effects on growth performance [131,132], while rapeseed supplementation has been shown to improve milk composition and enhance fatty acid profiles in lamb meat, contributing to improved nutritional quality [133,134].

Some patented formulations also include functional plant additives. For example, fennel seeds were included in one patented feed formulation [9], and experimental studies [135] have shown that fennel supplementation may improve growth performance and muscle development in lambs. Similarly, malt by-products (in patent [8]) and fermentation residues betel nut (in patent [8,9]) represent energy-rich and highly digestible feed components that support growth and metabolic demands, particularly during pregnancy, lactation, and finishing periods [136,137].

Overall, the inclusion of cereal grains, oilseed by-products, and plant-derived residues in patented MVC formulations reflects their dual function as nutrient sources and functional feed components. These ingredients provide energy, protein, fiber, and bioactive compounds that support rumen function, nutrient utilization, and animal productivity, while also serving as carriers for mineral and vitamin supplementation.

The widespread inclusion of plant-derived feed ingredients in patented MVC formulations reflects their importance not only as nutritional substrates but also as functional components that enhance mineral bioavailability, rumen fermentation, and overall metabolic efficiency in sheep.

## 6. Conclusions

Analysis of patented mineral–vitamin complexes for pregnant ewes and lambs demonstrates that their formulations are generally consistent with physiological requirements and current knowledge of ruminant metabolism. Essential macroelements and trace elements, together with vitamins A-, D-, E-, C-, K-, and B-group vitamins, support mineral homeostasis, energy metabolism, antioxidant defense, and immune function. The use of organic, inorganic, and nanoscale mineral forms reflects efforts to improve bioavailability, while functional additives such as probiotics, phytocomponents, and amino acids enhance nutrient utilization by modulating rumen microbial activity. The growing use of plant-derived bioactive compounds also reflects a shift toward safer, more sustainable alternatives to antibiotic growth promoters. Table 2 presents analytical data on the composition of patented mineral–vitamin complexes, with patent references indicated in the top horizontal row and the “+” symbol denoting the presence of specific components.

This table has practical significance, as it provides a useful reference for the rational formulation and optimization of mineral–vitamin feed additives for pregnant ewes and lambs.

Mineral and vitamin complexes function as synergistic multicomponent systems in which coordinated interactions between minerals, vitamins, and functional additives regulate metabolic pathways, mineral homeostasis, and antioxidant defense. These interactions are essential for physiological adaptation during pregnancy and early development. Optimizing the composition, bioavailability, and balance of MVC components is therefore critical for improving metabolic stability, reproductive performance, and offspring viability in sheep.

## Figures and Tables

**Table 1:** Functional additives in patented MVCs.

Functional Additives	Component of MVC	Patent
Probiotics	Bacillus Licheniformis	[21]
	Bacillus Subtilis	[21]
	Lactobacillus	[14]
	Bifidobacterium	[14]
	Bacillus Titanium Zinc	[1,2,6,7]
	Rumensins^TM^	[3,18]
	Pfansteihl (L-Lactic acid)	[21]
Prebiotics	Oligosaccharide	[21]
	Tangerine Peel	[9]
	Orange Peels	[21]
Glycoconjugate	Glycoconjugate	[21]
Starch	Starch	[16]
Ferments	Cellulase	[10,12,14]
	Protease	[10]
	Composite Fermentation Bacteria	[1,2,6,7]
Antibiotics	Monensin Sodium	[1,2,3,6,7,16,19]
	Bacitracin Zinc	[1,2,6,7,19]
Urea	Urea	[3,10,13,22]
	Biuret With The Purity Of 90.0%	[11]
Urease Inhibitor	Urease Inhibitor	[16]
Aminothiol	Mercaptamine (cysteamine)	[9]
Amino Acids	Compound Amino Acid	[14,23]
	Methionine	[13,14,21]
	Lysine	[13,14,19,21]
	Threonine	[14]
	Glutamic Acid	[17]
	Class Propylhomoserin	[17]
Yeast	Active Dry Yeast	[12,14,19]
Omega-3	Cod liver oil	[4,5]
Protein	Mulberry Leaf	[23]
	Little Peptide Albumen	[21]
	Casein	[21]
Phytocomponents	Plant extracts	[9,18]
	Chinese herbal medicine additive	[14,23]
	Subprostrate sophora, chrysanthemum, pseudo-ginseng, eclipta alba, astragalus membranaceus, glossy privet fruit, almonds, and bistort rhizome	[21]

**Table 2:** The composition of patented mineral–vitamin complexes.

		[1]	[2]	[3]	[4]	[5]	[6]	[7]	[8]	[9]	[10]	[11]	[12]	[13]	[14]	[15]	[16]	[17]	[18]	[19]	[20]	[21]	[22]	[23]
Minerals and microelements	Ca	+	+	+	+	+	+	+			+	+	+	+	+	+			+	+	+	+	+	+
	Zn	+	+	+	+		+	+	+		+	+	+	+	+		+	+			+	+		
	Mg						+	+	+		+			+			+	+		+	+			
	P	+	+				+	+	+		+									+				
	Na	+	+	+	+	+	+	+					+	+		+		+	+	+	+		+	+
	Se	+	+	+			+	+	+		+	+	+	+	+		+	+		+	+	+		
	Fe	+	+	+	+		+	+	+		+	+	+	+	+		+	+		+	+	+		
	Cu	+	+	+	+		+	+	+		+	+	+	+	+		+	+			+	+		
	Mn	+	+	+			+	+	+		+	+	+	+	+		+				+			
	K			+					+		+	+	+	+						+	+	+		
	I														+		+	+						
	Co	+	+	+			+	+	+		+	+	+	+	+		+	+						
	Cr									+									+					
	Clay			+						+	+		+				+		+					
	REE									+									+					
Vitamins	Multi-vitamins			+	+										+	+				+	+	+		
	Vitamin A				+	+			+		+	+	+	+	+	+	+	+				+		
	Vitamin D3					+			+		+	+	+	+	+	+	+	+				+		
	Vitamin E	+	+		+		+	+	+		+	+	+	+	+		+	+				+		
	Vitamin K				+							+			+							+		
	Vitamin C	+	+				+	+														+		
	Vitamin B1										+				+			+		+		+		
	Vitamin B2	+	+				+	+																
	Vitamin B6	+	+				+	+														+		
	Vitamin B12	+	+				+	+														+		
	Vitamin B5	+	+																					
	Vitamin B9										+													
	Vitamin B7										+													
	Vitamin B3								+		+							+						
	Vitamin B4																					+		
Functional additives	Omega-3				+	+																		
	Aminophylline																					+		
	Tryptophan																			+				
	Fattening feed																				+			
	Probiotics	+	+				+	+							+							+		+
	Prebiotics			+						+									+			+		
	Starch																+							
	Glycoconjugate																					+		
	Ferments	+	+				+	+			+		+		+									
	Antibiotics	+	+	+			+	+									+			+				
	Urea			+							+	+		+									+	
	Urease inhibitor																+							
	Aminothiol									+														
	Amino acid													+	+			+		+		+		+
	Yeast												+		+					+				
	Protein																					+		+
	Phytocomponents					+			+	+					+				+		+	+		+
Roughages	Roughages	+	+	+	+	+	+	+	+	+	+	+	+	+	+	+		+	+	+	+		+	+
	Residue	+	+	+	+	+	+	+	+	+			+		+	+		+	+	+	+		+	+

The symbol ‘+’ represents the presence of the specified mineral, vitamin, functional additive, or roughage within the respective patent.

## Data Availability

The data confirming the research data can be provided by the author for correspondence upon reasonable request. Public access to the data is restricted for correct use and interpretation.

## Abbreviations

The following abbreviations are used in this manuscript:

MVC	Mineral and Vitamin Complex
WIPO	World Intellectual Property Organization
FRAP	Ferric Reducing Antioxidant Power
NRC	National Research Council
MDA	Malondialdehyde
ABTS	2,2′-Azino-bis(3-ethylbenzothiazoline-6-sulfonic acid)
Zn-Met	zinc methionine
IgG	immunoglobulin G
IgM	immunoglobulin M
ZnT7	zinc transporter 7
ZnT8	zinc transporter 8
ZnT9	zinc transporter 9
ZIP7	Zrt- and Irt-like protein 7
GPx	Glutathione peroxidase
SOD	Superoxide dismutase
CAT	Catalase
REEs	Rare earth elements
DDGS	Dried distillers grains with solubles
